# Implementation of Multi-Criteria Decision-Making for Selecting Most Effective Genome Sequencing Technology

**DOI:** 10.3390/diagnostics15060665

**Published:** 2025-03-10

**Authors:** Ayse Arikan, Berna Uzun, Murat Sayan

**Affiliations:** 1 DESAM Research Institute, Near East University, TRNC, Mersin 10, 99138 Nicosia, Turkey; murat.sayan@kocaeli.edu.tr; 2 Department of Medical Microbiology and Clinical Microbiology, Near East University, TRNC, Mersin 10, 99138 Nicosia, Turkey; 3 Department of Medical Microbiology and Clinical Microbiology, Kyrenia University, TRNC, Mersin 10, 99320 Kyrenia, Turkey; 4 Operational Research Center in Healthcare, Near East University, TRNC, Mersin 10, 99138 Nicosia, Turkey; berna.uzun@neu.edu.tr; 5 Department of Mathematics, Near East University, TRNC, Mersin 10, 99138 Nicosia, Turkey; 6 PCR Unit, Research and Education Hospital, Kocaeli University, 41380 Kocaeli, Turkey

**Keywords:** genome detection, sequencing technologies, decision-making, fuzzy PROMETHEE

## Abstract

**Background/Objectives:** In recent years, molecular diagnosis has become increasingly critical in identifying human pathogens with unknown genes. **Methods:** An innovative approach, the fuzzy-based preference ranking organization method for enrichment evaluation (PROMETHEE) technique, one of the most effective multi-criteria decision-making (MCDM) methods, was used to evaluate criteria, including portability, generation type, max read/run, max output data/run, processing time/run, read length, accuracy, diagnostic sensitivity, test minimum loading volume, test cost/run, instrument cost, error rate, throughput capability, ability to sequence the large whole genome, small whole genome, and exome and large panel, mutation detection ability, whole-genome sequencing with single-stranded sequencing, and single-stranded sequencing accuracy, to determine the most suitable sequencing technology. **Results:** Based on the analysis, the Avidite Base Chemistry (ABC), Nanopore, and Illumina sequencing platforms sequentially emerged as the most favorable options based on their net flows of 0.0346, 0.0041, and 0.0003, respectively. **Conclusions:** Our findings provide important data to facilitate the selection of genome detection technologies. Through the use of innovative approaches, complex evaluations can be analyzed and the right choices can be made. Importantly, the technique has a degree of subjectivity, so varying conditions may lead to different findings.

## 1. Introduction

Infectious diseases are one of the most important causes of death worldwide. According to the World Health Organization’s global cause of death categorization, 55% of the analyzed 55.4 million deaths worldwide were due to cardiovascular, respiratory, and neonatal conditions [1]. It was estimated in another source that approximately 13.7 million (95% UI 10.9–17.1) people died due to infectious diseases [2]. While COVID-19, lower respiratory tract infections, and tuberculosis are among the top 10 causes of death globally, the highest mortality rates were reported for respiratory tract infections and bloodstream infections in 2021 [1,2]. Although many infections, such as human immunodeficiency virus (HIV), were not in the top 10 as of 2000, malaria, HIV/AIDS, and hepatitis B- and C-related cirrhosis were still the leading causes of death in low-income countries in 2019 [1]. These rates also varied by region and age. In sub-Saharan Africa, the mortality rate from bloodstream infections is 52.6 per 100,000, while in high-income countries, the rate is 37.7 per 100,000. Bloodstream infections are most common in adults aged 50–69, while children under 5 are the most affected by respiratory infections [2]. Moreover, the Global Burden of Disease (GBD) annually monitors the number of deaths and causes in different countries to ensure proper healthcare systems and measurements [3]. More than 607 billion highly standardized and comprehensive predictions from 12,000 people in 204 countries and territories are combined in estimating data on healthcare outcomes, systems, and global health trends [3]. In Turkey, COVID-19 (143.24 people/100,000 population) and lower respiratory diseases (15.66 people/100,000 population) were reported as the two most common causes of death in 2021 [4]. Tuberculosis in the Central African Republic and Kenya; COVID-19 in Tunisia, India, Indonesia, Iran, Brazil, Azerbaijan, Argentina, Albania, Jordan, Kazakhstan, Lebanon, and Mexico; malaria in Niger and Uganda; lower respiratory infections in Nigeria, South Sudan, and Togo; and HIV in Zimbabwe and Zambia were reported as the main causes of death due to infectious diseases in the GBD report [4].

The rapid and precise detection of disease-causing agents is essential as it reduces the mortality rate and accelerates treatment success. Since 1977, more advanced molecular and genetic approaches have been rapidly developed to provide more accurate and rapid diagnosis of infectious diseases with higher specificity and sensitivity than traditional diagnostic techniques [5]. Recently, various sequencing techniques and approaches have been widely implemented to characterize unknown microorganisms, identify subtypes, determine variations/mutations, and facilitate genomic studies [6,7,8,9,10]. Sequencing methods in clinical diagnosis and treatment are necessary, especially in molecular epidemiology studies, in examining mutation dynamics frequently encountered in infectious agents such as HIV, hepatitis B virus (HBV), cytomegalovirus (CMV), and hepatitis C virus (HCV); in monitoring antiviral activities in the treatment effectiveness of chronic patients infected with these agents; in patient management, including clade analyses in Monkeypox virus and human papillomavirus (HPV) typing; and in performing phylogenetic analysis and viral genotyping and/or subtyping for variant and mutation detection [11]. Closely monitoring chronic patients and performing these analyses using sequencing technologies are vital to increasing treatment success. Sequencing technologies have evolved and are categorized into three generations. The first generation is Sanger sequencing, which provides the basis for DNA sequencing. In contrast, the second generation consists of techniques that enable high-throughput sequencing by introducing massively parallel sequencing with platforms such as Illumina and Ion Torrent. The most advanced third-generation platforms, such as PacBio and Nanopore, offer long-read and single-molecule sequencing capabilities [9]. Despite advances in sequencing techniques, comparisons of the advantages, challenges, and limitations of sequencing are not focused on simultaneously. These comprehensive comparisons are not easy to make rationally; therefore, artificial intelligence- or mathematics-based innovative approaches are needed.

A subfield of decision science called ‘multi-criteria decision-making’ (MCDM) offers methodical guidance for simultaneously considering several factors for comparative analysis. MCDM approaches are frequently used to assess and contrast multiple alternatives in terms of a wide range of criteria in various professions, including engineering, finance, and healthcare [12]. Particularly, MCDM approaches are utilized in the healthcare industry to compare the performance of different diagnostic and therapeutic alternatives and guide decision-makers in making the right choice during treatment planning, since multiple factors are considered based on the physicians, patients, and their families [12]. Fuzzy logic has been incorporated with various MCDM methodologies. In this mathematical algorithm fusion, fuzzy set theory is an effective tool that enables the analysis of inaccurate and ambiguous information. The fuzzy preference ranking organization method for enrichment evaluation (fuzzy PROMETHEE) is one of the most common fuzzy MCDM methods that has been used in operational science [12]. Fuzzy PROMETHEE is founded on the idea of outranking, according to which alternative is ranked as outperforming another if it scores better on specified criteria [12]. Even though there are numerous DNA sequencing techniques, there is not much research that analyzes and contrasts these approaches, particularly by using the MCDM approach. The majority of research has compared the performance of DNA sequencing techniques based on just one criterion, like cost, efficacy, or safety, without considering other criteria that may diverge from the decision. This traditional mechanism might not offer a thorough assessment of the efficacy of the sequencing method; thus, it is necessary to consider several factors at once. In recent years, molecular diagnosis has become increasingly critical in identifying human pathogens with unknown genes. Accordingly, DNA sequencing techniques become more advanced and complex on a regular basis, depending on needs and technology. Therefore, we aim to simplify and enhance the selection of a wide range of DNA sequencing techniques by analyzing and comparing 15 DNA sequencing techniques using the MCDM approach, specifically fuzzy PROMETHEE, based on diverse parameters. Different countries can adapt the method applied in this study based on their needs and requirements, and the results of the study can assist engineers, scientists, and policymakers in making knowledge-based choices regarding the best-performing DNA sequencing techniques in diseases when there are many alternatives and criteria to consider.

The remainder of this work is divided as follows: the second section of the study expands on the considered parameters for comparing different sequencing methods and the applied decision analysis method and provides more details of how this analytical technique was applied, and the third section presents the results, whereas the fourth section discusses the findings of the study, with the conclusion of the work given in the fifth section.

## 2. Materials and Methods

This section contains detailed information about the PROMETHEE approach and data on selected sequencing technologies.

### 2.1. Selected Parameters of the Sequencing Technologies

The analysis involved the most high-quality and advanced models of each current and developing technique. Fifteen different sequencing techniques were evaluated using various criteria. The methods included were selected to cover various aspects of genetic sequencing technologies, offering the potential to serve a wide range of application areas. An innovative Avidite Base Chemistry technology (ABC) (AVITI, Element Biosciences, San Diego, CA, USA); long-read platforms including Single-molecule Real-time sequencing (SMRT); PacBio (Pacific Biosciences of California, Menlo Park, CA, USA); Nanopore sequencing (MinION, Oxford Nanopore Technologies, Oxford, UK); short-read platforms including Ion Torrent (Ion GeneStudio S5 Prime System 540 chip, Thermo Fisher Scientific, Waltham, MA, USA); Pyrosequencing (PyroMark Q96 Autoprep, Qiagen Inc., Germantown, MD, USA); Illumina semiconductor sequencing (Illumina HiSeq 3000/4000; Illumina Inc., San Diego, CA, USA) and GenapSys sequencing by synthesis (GenapSys Sequencer, Prayoga Lifesciences Inc., Karnataka, India); SOLID DNA sequencing (ABI SOLID, Applied Biosystems, Foster City, CA, USA) and Polony sequencing by ligation (Polonator G.007, Dover Motion, Boxborough, MA, USA); Sanger sequencing by incorporation of chain-terminating dideoxynucleotide (Sanger 3730 xl, Applied Biosystems, Foster City, CA, USA); Maxam–Gilbert sequencing by chemical degradation; and microfluidic DNA sequencing and CRISPR Cas sequencing technologies were considered. As a DNA Polymerase-based single-molecule sequencing technology, Helicos (Helicos Biosciences Corporation, Cambridge, MA, USA) was included (Table 1).

The criteria evaluated were as follows: portability (benchtop portable/benchtop small device/benchtop automated system); generation category (first/second/third generation); max read/run (KB/MG/GB); max output data/run (KB/MG/GB); processing time/run (hours/days); read length (short read/long read); accuracy (%); diagnostic sensitivity (%); test loading volume (minimum sample size in µL required for testing); test cost/run (minimum cost required for testing); instrument cost (minimum cost required for device); error rate (%); throughput capability (low/moderate/high/ultra-high); ability to sequence the large whole genome, small whole genome, and exome and large panel; mutation detection ability; whole-genome sequencing with single-stranded sequencing; and single-stranded sequencing accuracy. Detailed information about the dataset used in the analysis was gathered from the official product websites. We used data from the official websites of the sequencing technologies, and they were chosen because of their technical details and specifications [9,10,11,12,13,14,15,16]. To validate the data from the official website, the product details from the websites were compared based on specifications and peer-reviewed published studies. The accuracy of the data was also validated using an independent expert reviewer. We validated and standardized the data across different sources using measurement characteristics including read length/run, error rate, processing time/run, etc., to guarantee comparability. Afterward, we converted the linguistic scales into fuzzy numerical values.

### 2.2. Application of Fuzzy PROMETHEE in Selecting Sequencing Techniques

Fuzzy PROMETHEE is an extension of classical PROMETHEE that incorporates fuzzy logic to handle uncertainty and vagueness in decision-making processes. It is particularly useful in multi-criteria decision-making (MCDM) problems where subjective judgments and imprecise information exist. Fuzzy PROMETHEE allows decision-makers to express their preferences using linguistic terms, which are then converted into fuzzy numbers for ranking alternatives.

The technique consists of several key steps, including defining criteria, converting linguistic evaluations into fuzzy values, and calculating outranking flows to rank the alternatives based on their performance. In this study, a five-scale triangular fuzzy number system was used to convert the linguistic scale into numerical values, and the Yager index was applied to aggregate fuzzy numbers into single values for use in the PROMETHEE approach. Fuzzy sets, presented by Lotfi A. Zadeh in 1965, constitute a mathematical framework that allows the representation of uncertainty and imprecision numerically. Unlike classical (crisp) sets where elements either belong to a set or do not (binary logic), fuzzy sets allow elements to have degrees of membership ranging between 0 and 1. Considering that raw data are not always numerically precise or complete, appropriate methods such as fuzzy logic can be employed to handle uncertainty and imprecision. By classifying the data linguistically and converting them into fuzzy numbers, a more comprehensive and flexible analysis can be achieved, allowing for better decision-making even in the presence of incomplete or imprecise data.

The weighting of the criteria can also be defined linguistically, allowing experts to express their judgments using qualitative terms such as ‘Low’, ‘Medium’, or ‘High’. These linguistic terms can then be converted into fuzzy numerical values using appropriate membership functions, such as triangular or trapezoidal fuzzy numbers. This approach enhances flexibility in decision-making by accommodating subjective assessments and reducing the uncertainty associated with precise numerical weights.

In this study, the fuzzy PROMETHEE technique, one of the most effective MCDM methods, was chosen to identify the most suitable sequencing technology. By incorporating fuzzy sets [17], decision-makers can consider imprecise or ambiguous data in their evaluations [18]. This study utilized linguistic triangular fuzzy sets (a systematic way of representing data in a triangular shape where the lower and upper membership values are 0 and 1) to determine the features/criteria of the selected sequencing technologies and assign numerical weights to them. Subsequently, the defuzzification (i.e., converting and expressing the data, in linguistic forms like very high (VH), high (H), low (L), and very low (VL), to and in numbers) process was applied to convert the fuzzy data into numerical values, serving as a preprocessing step before applying the PROMETHEE approach. PROMETHEE allows decision-makers to rank alternatives based on multiple attributes. By offering various preference functions for calculating the preference index of each alternative, PROMETHEE enables more precise and sensitive rankings compared to other MCDM methods [18].

PROMETHEE is a model proposed by Brans in 1984. A recently used MCDM technique combines PROMETHEE with fuzzy logic to compare alternatives in an ambiguous environment [19]. In this approach, triangular fuzzy sets are used to process the linguistic data numerically as fuzzy sets/numbers.

In this study, we applied the fuzzy PROMETHEE approach for analysis, as it offers various types of preference functions to assess the superiority of each decision option for each criterion. This distinguishes it from other MCDA models, and the present approach also shows the advantages and disadvantages of each decision option that can be controlled by experts for the validation of results. A professor of microbiology and an associate professor of microbiology evaluated the criteria based on their experience in the field and categorized the criteria as ‘Very high’, ‘High’, ‘Moderate’, ‘Low’, and ‘Very low’, taking into account routine diagnoses and extraordinary situations such as hospital outbreaks or pandemics.

After data collection, linguistic data were converted into numerical form using fuzzy numbers, and then defuzzification was applied to convert fuzzy numbers to single numbers for the application of PROMETHEE for ranking alternatives. The Yager index [20] was used for the defuzzification process of the assigned fuzzy values to the linguistic scale (see Table 2) since it represents the center of the fuzzy sets, with the index taking into consideration every point in the sets to provide the defuzzified value. Assume that (*N, a, b*) represents a triangular fuzzy number/set, where *N* is the point with the maximum membership in the fuzzy set, *a* is the distance between *N* and the left bound of the set, and *b* is the distance between *N* and the right bound. The Yager index suggests the defuzzified point using the formula (*3N − a + b*)/*3*.

The Gaussian preference function was selected to obtain the priority values of the alternatives in degrees, providing preference values based on the standard deviation of the criteria rather than a binary 0 or 1.

The detailed process of the PROMETHEE approach is presented below [21].

Assuming that the decision matrix A, the dataset for the selection problem with multiple criteria, is obtained, and *P_j_*, the preferred function for the *j-th* criteria, is selected, $\pi \left(a_{t},a_{t^{\prime }}\right)$, the outranking relation values for every alternative pair $\left(a_{t},a_{t^{\prime }}\right)$ in A, should be calculated with Equation (1):

(1) $$\pi \left(a_{t},a_{t^{\prime }}\right)=\sum_{k=1}^{K}w_{k}.\left[p_{k}\left(f_{k}\left(a_{t}\right)-f_{k}\left(a_{t^{\prime }}\right)\right)\right],AXA\to \left[0,1\right]$$

where *k* denotes the number of criteria.

Then, the positive outranking flow ($\Phi^{+}\left(a_{t}\right)$), which shows the strength of the alternatives, and the negative outranking flow ($\Phi^{-}\left(a_{t}\right)$), which shows the weakness of the alternatives, should be calculated using Equations (2) and (3), respectively.

(2) $$\Phi^{+}\left(a_{t}\right)=\frac{1}{n-1}\sum_{\begin{array}{c} t^{\prime }=1\\ t^{\prime }\neq t \end{array}}^{n}\pi \left(a_{t},a_{t^{\prime }}\right)$$

(3) $$\Phi^{-}\left(a_{t}\right)=\frac{1}{n-1}\sum_{\begin{array}{c} t^{\prime }=1\\ t^{\prime }\neq t \end{array}}^{n}\pi \left({a_{t^{\prime }},a}_{t}\right)$$

The partial pre-order results of the alternatives can be obtained using the following statements:

The alternative $a_{t}$ has a higher rank than $a_{t^{\prime }}$ if

(4) $$\begin{array}{c} \Phi^{+}\left(a_{t}\right)\geq \Phi^{+}\left(a_{t^{\prime }}\right)\;\mathrm{and}\;\Phi^{-}\left(a_{t}\right)<\Phi^{-}\left(a_{t^{\prime }}\right)\\ \Phi^{+}\left(a_{t}\right){>\Phi }^{+}\left(a_{t^{\prime }}\right)\;\mathrm{and}\;\Phi^{-}\left(a_{t}\right)=\Phi^{-}\left(a_{t^{\prime }}\right)\\ \; \end{array}$$

$a_{t}$ has an equal rank to $a_{t^{\prime }}$ if

(5) $$\Phi^{+}\left(a_{t}\right)=\Phi^{+}\left(a_{t^{\prime }}\right)\mathrm{and}\;\Phi^{-}\left(a_{t}\right){\;=\Phi }^{-}(a_{t^{\prime }})$$

$a_{t}$ and $a_{t^{\prime }}$ are not comparable if

(6) $$\left\{\begin{array}{c} \Phi^{+}\left(a_{t}\right)>\Phi^{+}\left(a_{t^{\prime }}\right)\;\mathrm{and}\;\Phi^{-}\left(a_{t}\right)>\Phi^{-}\left(a_{t^{\prime }}\right)\\ \Phi^{+}\left(a_{t}\right)<\Phi^{+}\left(a_{t^{\prime }}\right)\;\mathrm{and}\;\Phi^{-}\left(a_{t}\right)<\Phi^{-}\left(a_{t^{\prime }}\right) \end{array}\right.$$

This process could also bring decision-makers to a point where choices cannot be compared. In such cases, the PROMETHEE II process will enable the decision-maker to obtain the net ranking results of the alternatives ($\Phi^{net}\left(a_{t}\right))$ using Equation (7):

(7) $$\Phi^{net}\left(a_{t}\right)=\Phi^{+}\left(a_{t}\right)-\Phi^{-}\left(a_{t}\right)$$

The ranking of the alternatives should be obtained in descending order based on $\Phi^{net}\left(a_{t}\right)$. The option that has the highest values should be considered the most appropriate or effective alternative under the selected criteria and priorities given for the criteria.

The weights of the selected criteria are shown in Table 2. None of the criteria have been assigned a low or very low weight based on the experts’ opinions when considering global health emergencies such as outbreaks and pandemics.

## 3. Results

The present study presents a complete ranking of DNA sequencing methods using the Fuzzy PROMETHEE technique. This ranking helps in understanding the relative merits and demerits of the selected sequencing technologies, which can be useful for research, clinicians, and industrial decision-makers. The sequencing techniques were ranked from the most preferred to the least preferred. According to the fuzzy PROMETHEE ranking, ABC sequencing ranked first with a $\Phi^{net}$ score of 0.0346, Oxford Nanopore Technology ranked second with a $\Phi^{net}$ score of 0.0041, and Illumina, Single Molecule Fluorescent Sequencing, ABI’s SOLID sequencing (SOLID v4), and Single-molecule Real-time sequencing ranked next with $\Phi^{net}$ scores of 0.0003, 0.0002, −0.0014, and −0.0016, respectively. The ranking results of all selected DNA sequencing platforms are presented in Table 3.

In Table 3, $\Phi^{+}$ indicates the positive flow (strength or outranking score), while $\Phi^{-}$ represents the negative flow (weakness or outranked score), both summarizing the performance of alternatives in the decision-making process. A higher $\Phi^{+}$ indicates better performance (greater strength in outranking others), whereas a lower $\Phi^{-}$ reflects weaker performance (less strength, being outranked by others). $\Phi^{net}$ represents the net flow (overall preference score), calculated as the difference between $\Phi^{+}$ (positive flow) and $\Phi^{-}$ (negative flow), offering a comprehensive measure of an alternative’s overall standing in the decision-making process.

According to the net values, a gradual decrease was detected between each technique. Avidite Base Chemistry sequencing has a significantly higher $\Phi^{net}$ value than other methods, indicating that it has particularly strong advantages in key areas. Furthermore, Illumina NextSeq 2000 and Single Molecule Fluorescent Sequencing have almost identical $\Phi^{net}$ values, suggesting that their overall performance across all criteria is comparable. They may share similar strengths and trade-offs, making them nearly interchangeable. Thus, beyond ranking, the magnitude of differences in $\Phi^{net}$ values provides insight into how competitive the methods are with each other. Small differences in $\Phi^{net}$ suggest close competition, while large differences indicate a clear preference for certain methods over others.

**Figure 1:**
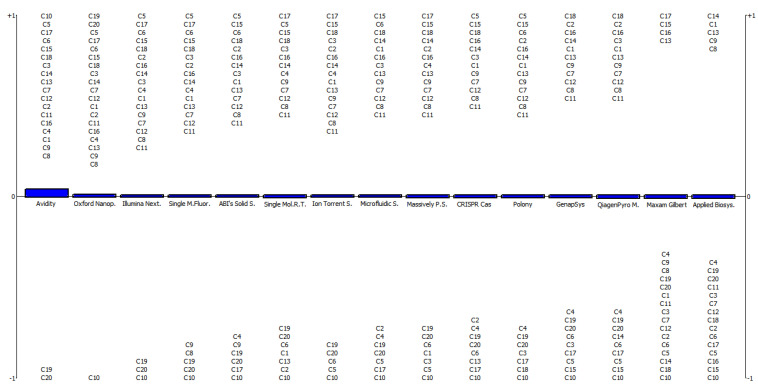
PROMETHEE technique results. Abbreviations: C—criteria; C1—machine design; C2—type of sequencing; C3—generation type; C4—DNA or RNA sequencing; C5—max read length/run; C6—max output data/run; C7—processing time/run; C8—accuracy; C9—diagnostic sensitivity; C10—cost per instrument; C11—cost/run; C12—cost per instrument; C13—error rate; C14—throughput; C15—large whole-genome sequencing (human, plant, animal); C16—small whole-genome sequencing (microbe, virus); C17—exome and large panel sequencing (enrichment-based); C18—loading volume; C19—whole genome with single-stranded sequencing; C20—single-stranded sequencing accuracy.

The positive and negative aspects of each selected DNA sequencing option are presented in Figure 1. Features above the 0 threshold represent the positive aspects of the respective option, while features below the 0 threshold indicate the negative aspects. The results were derived from the Decision Lab program. These results provide critical insights that can aid decision-makers in identifying opportunities, mitigating risks, and making data-driven decisions.

### Sensitivity Analysis

Sensitivity analysis was performed to assess the reliability and robustness of our findings. It involved executing experiments with varying criteria weights and examining the resultant rankings. This sensitivity analysis elucidates the relative importance of different criteria when modified, thus enhancing our understanding regarding their influence on the final results. We changed the criteria weights of accuracy, diagnostic sensitivity, cost per instrument, cost/run, throughput, and loading volume from Very High to High without altering other criteria weights. The results of the analysis show that ABC sequencing still ranks first, followed by Oxford Nanopore sequencing, which shows the robustness of our results. Table 4 presents the results of the sensitivity analysis.

As observed in Figure 1, the validity sequencing method ranked first due to factors such as value-added machine design (C1), the precision of the machine (C8), the throughput of the machine (C14), its high diagnostic sensitivity (C9), and it being well suited for handling large whole-genome sequencing assignments for extensive detailed evaluation (C15), making it most preferable for sample-based workflows that require high throughput and high precision. Furthermore, some of the advantages of Oxford Nanopore Technology is its performance in the long-read sequencing mode (C5) and its versatility in terms of compatibility with both DNA and RNA sequencing (C4). The fairly high positive flow of 0.0064 reveals its strength in terms of flexibility and portability, which makes it suitable for use in real-world applications as well as field sequencing. However, its high cost (C10) compared to Avidite Base Chemistry sequencing slightly reduced its preference score. Illumina NextSeq, Single Molecule Fluorescent Sequencing, ABI’s SOLID sequencing, and Single-molecule Real-time sequencing appeared in the mid-tier of the ranking, although, in a comparison between them, it appears that Illumina occupies a dominant position in the sequencing market since it contributes an appropriate balance between cost (C11) and accuracy (C8), which ensures its suitability for straightforward laboratory use.

Single Molecule Fluorescent Sequencing yielded comparable performance and emerged as superior in terms of processing time (C7) and loading capability (C18). However, it had a mildly lower diagnostic sensitivity (C9) compared to ABC and Oxford Nanopore systems which limited its final ranking. The fairly low-level technologies, including Ion Torrent semiconductor sequencing, Microfluidic Sanger sequencing, and massively parallel signature sequencing, ranked lower owing to weaknesses in sequencing type (C2), short-read-length sequencing and output run (C5 and C6), affordability (C10), applicability to the whole genome with single-stranded sequencing, and accuracy (C19 and C20). Additionally, the Maxam–Gilbert method and the Applied Biosystems 3730 were in the last rank as they are no longer considered advanced due to their high operational costs (C11), relatively low throughput (C14), and manual processes (C7). Due to their poor scalability and inability to meet present-day sequencing demands, they are unsuitable for contemporary genomic study.

## 4. Discussion

Managing the heavy workload in the healthcare system is of great importance in the clinical management of patients, and in recent years, sequencing techniques have been applied for this purpose. Various factors, including the speed of diagnosis, cost, size of the genetic material to be analyzed, and characteristics of the patient and disease, play a crucial role in determining the most suitable sequencing method. Choosing the right approach speeds up the diagnosis process and makes treatment and monitoring processes more effective.

Fuzzy PROMETHEE enables multi-criteria decision-making by integrating diverse factors such as diagnostic sensitivity, error rates, throughput, and cost within a unified framework. Unlike traditional approaches, it accommodates uncertainty through linguistic scales and fuzzy numbers, allowing for a more nuanced comparison of sequencing technologies. Moreover, the main feature of Fuzzy PROMETHEE is its ability to provide a comprehensive and balanced evaluation process. In cases where there are uncertainties and contradictions between criteria, it effectively eliminates contradictions through preference functions and fuzzy logic. This allows more informed and reliable results to be achieved, especially in complex decision-making problems. However, there are limitations of this study that should be pointed out: In the fuzzy logic method, the importance of criteria is determined according to the expertise, priorities, and perspective of the decision-makers. This may lead to different findings under different conditions. As a result, in this study, we aimed to incorporate a large dataset, diverse experts in decision-making, and alternative weighting techniques to enhance the method’s robustness. Therefore, we implemented fuzzy PROMETHEE, a method widely utilized in the medical field [18,19,20,21,22], to guide the selection of the most appropriate and effective sequencing techniques for clinical use. Our findings revealed that innovative sequencing chemistry, specifically Avidite Base Chemistry sequencing technology, was the most favorable system, with the ability to provide higher accuracy even in variant detection with lower reagent consumption [23].

Due to a reduction in tandem repeats and homopolymers, this new approach can obtain more accurate reads using fewer chemicals compared to Illumina or the other long-read platforms ranked third [13,23]. Moreover, a special property that is only available in ABC technology is that it is possible to upgrade to a later version by updating the software without updating the hardware. This allows the system to be renewed without the need to purchase a new device, which enables efficiency in terms of cost and time.

In this ranking, Nanopore was in second place. Direct oligonucleotide sequencing with nanopore technology enables the sequencing of short single-stranded unamplified DNA fragments without the need for the complimentary step or second-strand synthesis by performing a single annealing step before library preparation. This system provides qualified analysis with minimal steps even at low viral loads, increasing cost-effectiveness similarly to the ABC system [24]. In addition, since both techniques can provide results overnight, they may be preferred for patient follow-up in patients with chronic infections, mutation analysis in patients treated with antivirals, and the monitoring of infectious disease outbreaks. This allows for rapid diagnosis to be made and action to be taken. With these features, it is no surprise that the $\Phi^{net}$ scores of the ABC and Nanopore technologies were higher than those of other techniques in the ranking.

Meanwhile, in terms of the implications for lower-ranked technologies, Single Molecule Fluorescent Sequencing offers cost-effectiveness, a satisfactory processing duration, and suitableness for laboratory use. Such technologies are promising solutions in low-resource environments but have limitations like less diagnostic sensitivity. Subordinate technologies like Maxam–Gilbert and ABI’s SOLID sequencing have been criticized for their high operational costs, outdated systems, and limited scalability. The authors propose improvements, such as automation, cost reduction, and the integration of modern features, to improve their applicability in clinical and research settings. They also discuss the wider implications of using mid- and lower-tier technologies in specific contexts, such as educational applications or budget-constrained pilot research. Recommendations for manufacturers and researchers to improve critical limits like error rates and throughput will inform future progress.

Our findings showed that Single Molecule Fluorescent Sequencing was ranked in the sixth position. This technology does not require sample preparation, ligation, or PCR amplification, and avoids the GC content and size biases observed in other technologies. DNA is simply sheared, poly(A)-tailed, and hybridized to a flow cell surface containing oligo(dT) for the parallel sequencing of billions of molecules. This process also requires significantly less material than other technologies; however, due to the inadequacy of some of its properties including accuracy, sensitivity, cost-effectiveness, lower throughput, etc., compared to ABC, Nanopore, and Illumina, it ranked lower on the list [14].

In recent years, microfluidic devices and CRISPR/Cas technology have been developed to improve test performance with reduced sample volume, reagent consumption, cost, and high throughput [15,25]. Although these systems are portable and well designed, there are some concerns with them. For microfluidic devices, applications may be limited by chip size and manufacturing techniques. Cross-contamination is also possible due to the difficulty of achieving complete isolation between microwells. In addition, sample retrieval from microwell chips is difficult [15]. Moreover, the biggest problems with CRISPR/Cas technology are an off-target effect, the inability to deliver material into cells, and gene editing activity itself [26]. Although these new approaches offer significant hope for medical diagnosis, further effort may still be required to address these challenges.

ABC, Nanopore, Illumina, and PacBio are known as high-throughput, cost-effective, faster NGS platforms with broad utility capabilities including infection control applications as well as genetic diversity, gene expression, and microbial diversity applications [8,24]. Various NGS platforms are implemented in different fields and studies due to their distinct benefits and limitations. However, the underlying chemical differences may affect their read length, error rate, and throughput [26]. Therefore, there are still conflicts as to which technique would be most appropriate for the specified purposes [26]. The fuzzy PROMETHEE model used in this study allows decision-makers to prioritize according to the purpose of use, priority levels, and country conditions and enables them to determine the most effective technique to be used in clinics. This approach enabled us to grade and categorize sequencing technologies according to the priorities of healthcare systems and requirements by evaluating multiple criteria and considering uncertainty in the assessment. However, it is necessary to state that the ranking is heavily dependent on the weights assigned to each criterion. These weights could be adjusted, hence the importance of having the analysis fit the different objectives of the specific application. That is, this approach may vary and yield different results depending on the circumstances of different countries and hospital facilities. Advances in sequencing platforms have been made to develop more reliable and accessible systems compared to conventional systems, to integrate samples into automated miniature devices to generate the entire genome without any manual preparation, and to increase the use of diagnostic sequencing platforms worldwide. However, these advances can lead to confusion and unsuitable selections. Therefore, through the integration of interdisciplinary studies, confusion can be eliminated and the right decision can be made by using the fuzzy PROMETHEE model.

## 5. Conclusions

In conclusion, our findings provide important data to facilitate the selection of genome detection technologies for clinical use. MCDM techniques—particularly, fuzzy PROMETHEE—which enable experts to evaluate complex datasets have proven effective in offering users concise insights into gene sequencing technologies by ranking ABC Sequencing Chemistry and Oxford Nanopore as techniques that outperform the others which are highly versatile for many applications. However, intermediate-level technologies such as Illumina and Single Molecule Fluorescent Sequencing remain comparatively affordable options for low-budget or specific applications. The fact that there are no major differences between the net values of each technique indicates that other techniques with lower net values can also be used in conditions that are inaccessible to techniques such as ABC or Nanopore. This sequencing can facilitate operational efficiency and strategic decision-making. By providing a structured approach to technology selection, sequencing can facilitate informed decisions, improve outcomes, and drive innovation in genomic research and diagnostics. The application of fuzzy PROMETHEE in different sectors may allow countries to organize their infrastructure and control epidemics in future emergencies such as natural disasters. Importantly, the technique has a degree of subjectivity, so varying conditions may lead to different findings. Moreover, multicenter studies reveal important features of new techniques for disease detection and may guide manufacturing companies in the production of technologies tailored to specific needs.

## Figures and Tables

**Table 1:** Read lengths and mechanisms of sequencing technologies.

Generation	Sequence Technology	Read Type	Sequencing Method
First Generation			
	Maxam–Gilbert	Short read	Chemical degradation
	Sanger 3730 xl	Short read	Dideoxy chain-termination sequencing
Second Generation			
	Ion GeneStudio S5 System	Short read	Synthesis
	Illumina HiSeq 3000/4000	Short read	Synthesis
	Helicos Biosciences	Short read	Synthesis
	ABI SOLID	Short read	Ligation
	Polonator G.007 Sequencing	Short read	Ligation
	PyroMark Q96 Autoprep	Short read	Pyrosequencing
	GenapSys Sequencer	Short read	Electrical-based sequencing
	Microfluidic DNA Sequencing	Short read	Microfluidic-based sequencing
	Single Molecule Fluorescent Sequencing	Short read	Fluorescence-based single-molecule protein sequencing
	CRISPR Cas Sequencing	Short read	CRISPR targeted sequencing
Third Generation			
	MinION, Oxford Nanopore	Long read	Nanopore sequencing
	PacBio	Long read	Single-molecule DNA sequencing
	Avidite Base Chemistry	Short read	Binding chemistry

**Table 2:** Fuzzy scale and the selected importance weights of the criteria.

Linguistic Scale	Criteria
VH/(0.75, 1, 1)	Type of sequencing, max read length/run, max output data/run, processing time/run, accuracy, diagnostic sensitivity, cost per instrument, error rate, whole genome with single-stranded sequencing, single-stranded sequencing accuracy
H/(0.50, 0.75, 1)	Machine design, generation type, cost/run, throughput, large whole-genome sequencing (human, plant, animal), exome and large panel sequencing (enrichment-based), mutation detection
M/(0.25, 0.50, 0.75)	DNA or RNA sequencing, loading volume, small whole-genome sequencing (microbe, virus)
L/(0, 0.25, 0.50)	-
VL/(0, 0, 0.25)	-

Abbreviations: VH: very high; H: high; M: moderate; L: low; VL: very low.

**Table 3:** Ranking of DNA sequencing techniques with fuzzy PROMETHEE.

Rank	Alternative	$\Phi^{net}$	$\Phi^{+}$	$\Phi^{-}$
1	Avidite Base Chemistry Sequencing	0.0346	0.0348	0.0002
2	Oxford Nanopore Sequencing	0.0041	0.0064	0.0022
3	Illumina NextSeq 2000	0.0003	0.0028	0.0025
4	Single Molecule Fluorescent Sequencing	0.0002	0.0029	0.0027
5	ABI’s SOLID Sequencing	−0.0014	0.0019	0.0033
6	Single-Molecule Real-Time Sequencing	−0.0016	0.0020	0.0036
7	Ion Torrent Semiconductor Sequencing	−0.0016	0.0018	0.0035
8	Microfluidic Sanger Sequencing	−0.0021	0.0015	0.0036
9	Massively Parallel Signature Sequencing	−0.0022	0.0016	0.0039
10	CRISPR Cas	−0.0025	0.0013	0.0038
11	Polony Sequencing	−0.0028	0.0016	0.0045
12	GenapSys Sequencing	−0.0047	0.0007	0.0054
13	Qiagen PyroMark Q48 Autoprep	−0.0061	0.0006	0.0067
14	Maxam–Gilbert Method	−0.0063	0.0010	0.0073
15	Applied Biosystems 3730	−0.0078	0.0002	0.0081

Abbreviations: $\Phi^{net}$—net flow value; $\Phi^{+}$—positive outranking flow; $\Phi^{-}$—negative outranking flow.

**Table 4:** Sensitivity analysis ranking results.

Rank	Alternative	$\Phi^{net}$	$\Phi^{+}$	$\Phi^{-}$
1	Avidity Base Sequencing	0.0377	0.0380	0.0002
2	Oxford Nanopore Sequencing	0.0045	0.0069	0.0025
3	Single Molecule Fluorescent Sequencing	0.0003	0.0031	0.0028
4	Illumina NextSeq 2000	0.0003	0.0031	0.0028
5	ABI’s SOLID Sequencing	−0.0015	0.0021	0.0036
6	Single-Molecule Real-Time Sequencing	−0.0018	0.0021	0.0039
7	Ion Torrent Semiconductor Sequencing	−0.0018	0.0020	0.0038
8	Microfluidic Sanger Sequencing	−0.0023	0.0016	0.0039
9	Massively Parallel Signature Sequencing	−0.0024	0.0018	0.0042
10	CRISPR Cas	−0.0028	0.0014	0.0041
11	Polony Sequencing	−0.0031	0.0018	0.0049
12	GenapSys Sequencing	−0.0051	0.0008	0.0059
13	Qiagen PyroMark Q48 Autoprep	−0.0067	0.0007	0.0073
14	Maxam–Gilbert Method	−0.0068	0.0011	0.0079
15	Applied Biosystems 3730	−0.0086	0.0002	0.0088

## Data Availability

The datasets generated and analyzed during the current study are available from the corresponding author on reasonable request.
